# Supplementary material for: Highly bright perovskite light-emitting diodes enabled by retarded Auger recombination
Source: Nat Commun. 2025 Jan 22;16:927. doi: 10.1038/s41467-025-56001-x (PMC11754759; doi:10.1038/s41467-025-56001-x)
Supplement: Supplementary file 1 — Supplementary Information [file 41467_2025_56001_MOESM1_ESM.pdf]

# Highly bright perovskite light-emitting diodes enabled by retarded Auger recombination

Zhiqi Li[1, 2, 3, #], Qi, Wei[4, #], Yu Wang[1], Cong Tao[5], Yatao Zou[5], Xiaowang Liu[5], Ziwei Li[6], Zhongbin Wu[5], Mingjie Li[4*], Wenbin Guo[3*], Gang Li,[2*] Weidong Xu[5, 7*], Feng Gao[1*]

[1]Department of Physics, Chemistry and Biology (IFM), Linköping University, Linköping SE-58183, Sweden.

[2]Department of Electrical and Electronic Engineering, Photonic Research Institute (PRI), Research Institute of Smart Energy (RISE), The Hong Kong Polytechnic University, Hung Hom, Kowloon, Hong Kong, China.

[3]State Key Laboratory of Integrated Optoelectronics, College of Electronic Science and Engineering, Jilin University, 2699 Qianjin Street, Changchun 130012, People's Republic of China.

[4]Department of Applied Physics, The Hong Kong Polytechnic University, Hung Hom, Kowloon, Hong Kong, China.

[5]Frontiers Science Center for Flexible Electronics, Institute of Flexible Electronics (IFE), Northwestern Polytechnical University, Xi'an, People's Republic of China.

[6]Hunan Institute of Optoelectronic Integration, College of Materials Science and Engineering, Hunan University, Changsha 410082, China.

[7]Hunan Institute of Optoelectronic Integration, College of Materials Science and Engineering, Hunan University, Changsha 410082, China.

[#] These authors contributed equally.

**Supplementary Note 1**

The theoretical radiative emission efficiency in 3D perovskites can be given by the equation:

$$\eta(\mathrm{n}) = \frac{k_{\mathrm{ex}}+k_2\mathrm{n}}{k_{\mathrm{ex}}+k_{\mathrm{tr}}+k_2\mathrm{n}+k_3\mathrm{n}^2} \qquad (1)$$

where n is the carrier density, $\mathrm{k_{ex}}$ is the first-order radiative exciton recombination constant, $k_{\mathrm{tr}}$ is the monomolecular trap-assistant recombination constant, $k_2$ is the bimolecular recombination constant, and $k_3$ is the three-body Auger recombination constant, respectively. The emission efficiency is strongly dependent on carrier density and influenced by the trade-off of first-order radiative exciton recombination, trap-assistant recombination, bimolecular recombination and Auger recombination. Both exciton recombination and trap-assisted nonradiative recombination possess monomolecular recombination feature, named as the first-order recombination (the first-order recombination constant: $k_1$, containing both excitonic and trap-assisted process). Under low excitation intensity, emission efficiency depends on the competition between first-order exciton recombination and trap-assistant nonradiative recombination. Radiative bimolecular recombination gradually dominates over the monomolecular process with increased n. Furthermore, Auger recombination becomes very prominent and dominant at even higher carrier density.

In 3D perovskites, the overall charge-carrier dynamics can be described by the following rate equation:

$$\frac{\mathrm{dn}}{\mathrm{dt}} = -k_1\mathrm{n} - k_2\mathrm{n}^2 - k_3\mathrm{n}^3 \qquad (2)$$

The $k_1$ was experimentally determined from the TRPL kinetics under low pump fluences, where the first-order recombination was dominant and the high-order recombination contribution was negligible. We employed numerical integration to further simulate the kinetics for each sample by setting $k2$ and $k3$ as free fitting parameters to constrain the fitting. By conducting transient absorption measurement with various pump fluences, the bleach kinetics under different pump fluences are analyzed using a global fitting procedure to simultaneously simulate the data. The results reveal that the bimolecular and trimolecular recombination constants are decreased. Especially, the trimolecular Auger recombination constants of FCT-films are more than one-order-of magnitude lower than F-films, confirming that the Auger recombination rate has been notably suppressed.

**Supplementary Note 2**

We prepare a series of perovskite films with different surface coverage as a case study for further validating our findings. Specifically, formamidinium lead tri-iodide ($FAPbI_3$) films prepared from the precursors with the molar ratio of formamidinium iodide (FAI): lead iodide ($PbI_2$) =2.0:1, 2.4:1 and 2.8:1 in DMF were carefully studied, referred to as FAI-2.0, FAI-2.4. FAI-2.8 respectively unless otherwise stated. To exclude the influence of crystal defects and thus allow us to focus on the effects of morphology, a small amount of 5-ammonium valeric acid iodide (5AVAI) is incorporated into the precursors for defect engineering. Supplementary Figure 12 displays the morphological characteristics of perovskite films recorded by scanning electron microscopy (SEM). With reducing FAI contents, we observe that the surface coverage is improved and the grain size is simultaneously decreased. Among others, the FAI-2.0 films show the best surface coverage, although the pin-holes are still visible.

To establish the correlations between coverage and device performance, we proceed to fabricate the PeLEDs dependent on these 3D perovskite thin-film emitters. Among others, FAI-2.4 devices show the highest peak EQE of 21.3%, compared to 20.9% of FAI-2.0 and 18.1% of FAI-2.8 devices (Supplementary Figure 12 c and d). The best peak EQEs of FAI-2.4 can be assigned to the large grain size and the passivating ligands from excess FAI, while the decreased performance of FAI-2.8 is assigned to the reduced electronic properties of perovskite films from increased organic halides as the previous reports. Notably, despite slightly lower peak EQE values, the devices with the best surface coverage of perovskites (FAI-2.0 devices) shows the most efficient current injection and the best performance at the high current densities, thus resulting in the largest peak radiance (Supplementary Figure 12e).

One of the major concerns regarding the above conclusion is that the excess insulating FAI in perovskite thin-films could inhibit the carrier injection and thus deteriorate to the device performance at high current densities. We thus further control the surface coverage of the samples with identical precursor stoichiometry (FAI-2.0) by using solvent annealing strategy, that is, with DMF vapour during thermal annealing. As expected, the films with solvent annealing treatment gives rise to lower surface coverage and thus worse device performance at high current densities (Supplementary Figure 13). Additionally, we believe that different grain sizes arise from different precursor stoichiometry and thin-film deposition technique are not the key factor determining device characteristics in terms of current injection and EQEs at high current densities, as a large grain size usually results in slower Auger recombination and better charge carrier mobility. Both are not in line with the device results in the current cases. As such, we conclude that discrepancies in surface coverage determines the different device characteristics.

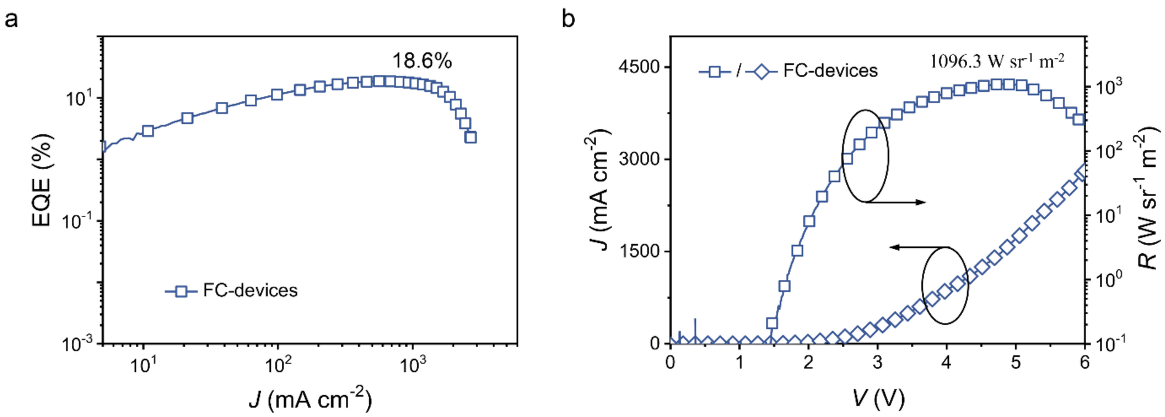


**Supplementary Figure 1. Performance of FC-PeLEDs. a**, EQE plotted against current density (EQE-*J*) for FC-devices. **b**, Dependence of current density and radiance on the voltage (*J*/*R*-*V*) of FC-devices. Here, FC denotes CsI based devices.

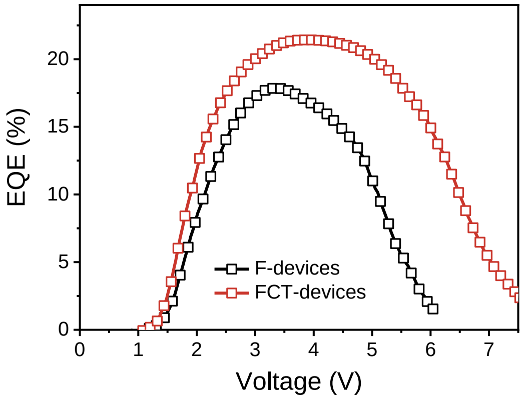


**Supplementary Figure 2. Performance of PeLEDs.** EQE plotted against voltage of F- and FCT-devices.

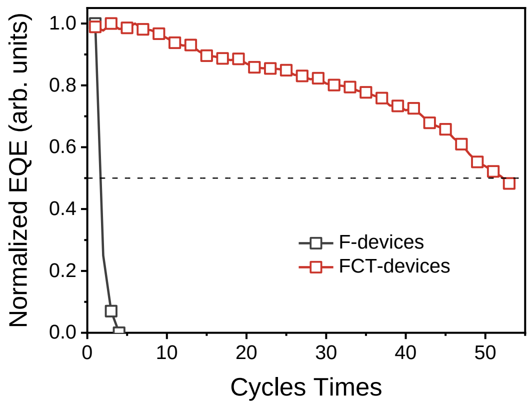


**Supplementary Figure 3. Reliability of PeLEDs upon voltage scans.** Normalized peak EQE as a function of cycle times of PeLEDs.

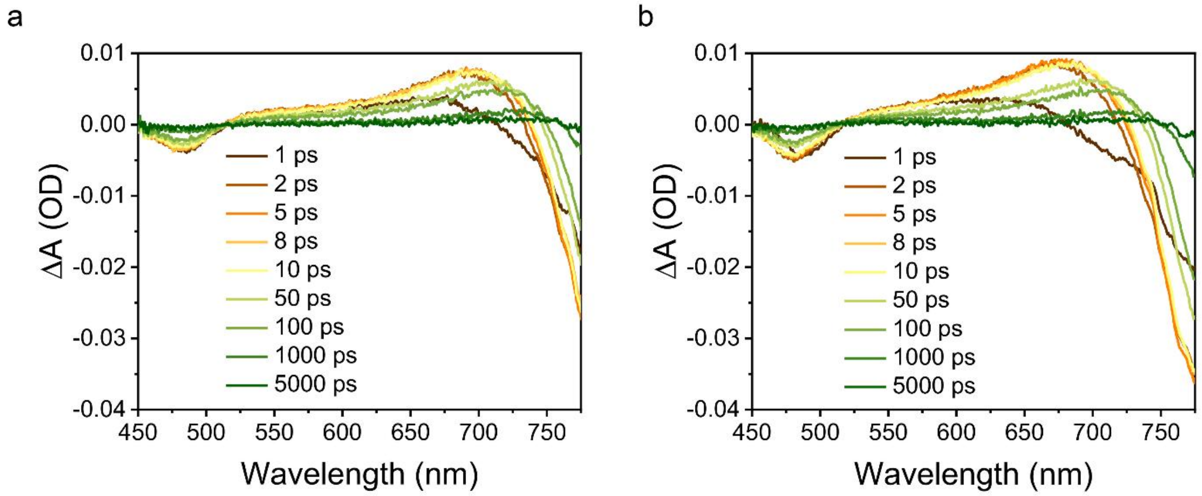


**Supplementary Figure 4. Transient absorption (TA) spectra for perovskite films. a**, F-films. **b**, FCT-films. All the samples were excited with 3.10 eV photons.

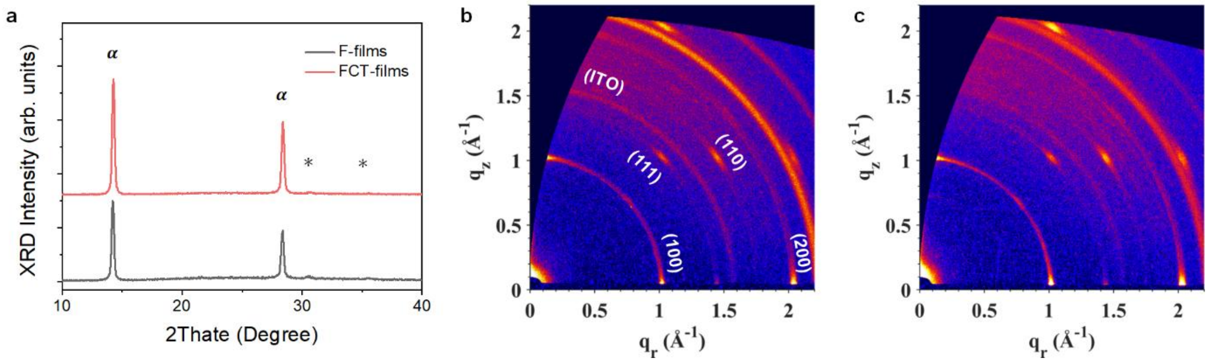


**Supplementary Figure 5. Crystallization properties of Perovskite films. a,** X-ray diffraction (XRD) patterns for F- and FCT-films. α and * denote the identified diffraction peaks corresponding to the α-$FAPbI_3$ and ITO. **b-c,** and grazing incidence wide angle X-ray scattering (GIWAXS) measurements for F- (b) and FCT-films (c).

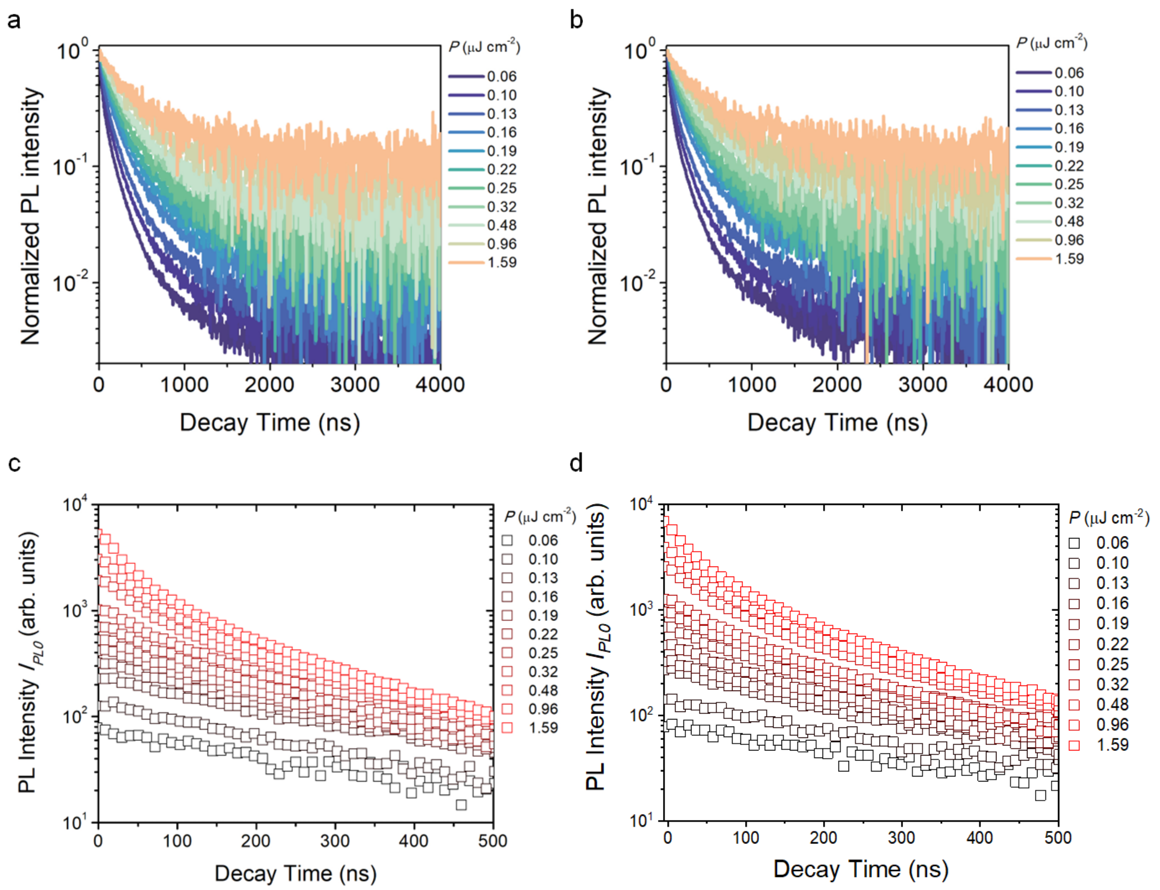


**Supplementary Figure 6. Recombination dynamics of perovskite films.** The power-dependent time-resolved spectra (**a**, **b**) and relevant PL intensity as a functional of decay time (**c**, **d**) for F- (**a**, **c**) and FCT-films (**b**, **d**). TRPL measurements for glass/ITO/ZnO/PEIE/ perovskite samples under 400 nm pulsed laser excitation.

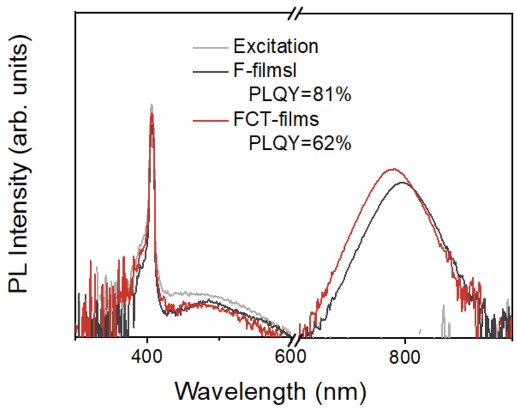


**Supplementary Figure 7. The photoluminescence characterizations of perovskite films.** The photoluminescence quantum yields (PLQYs) of F- and FCT-films.

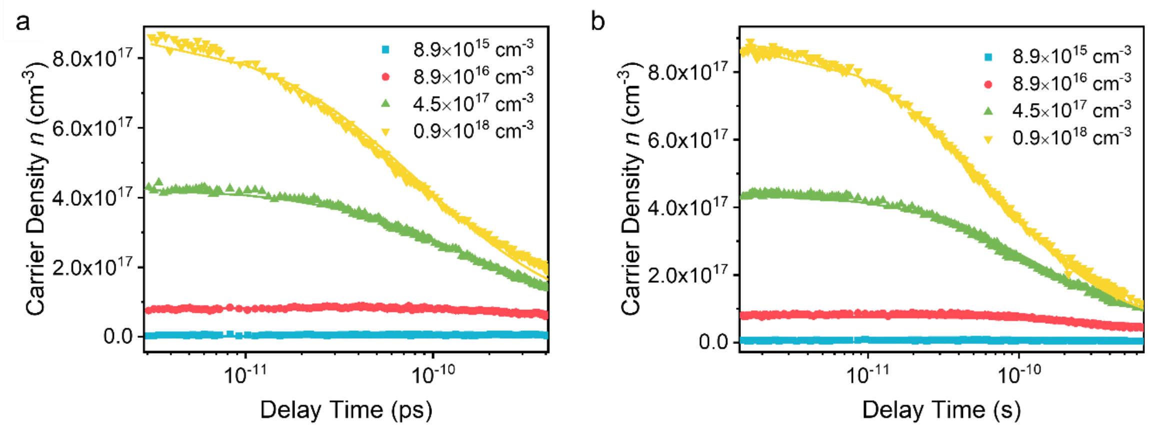


**Supplementary Figure 8. Carrier dynamics measurements of perovskite films.** Carrier dynamics probed at band-edge for (**a**) F-films and (**b**) FCT-films.

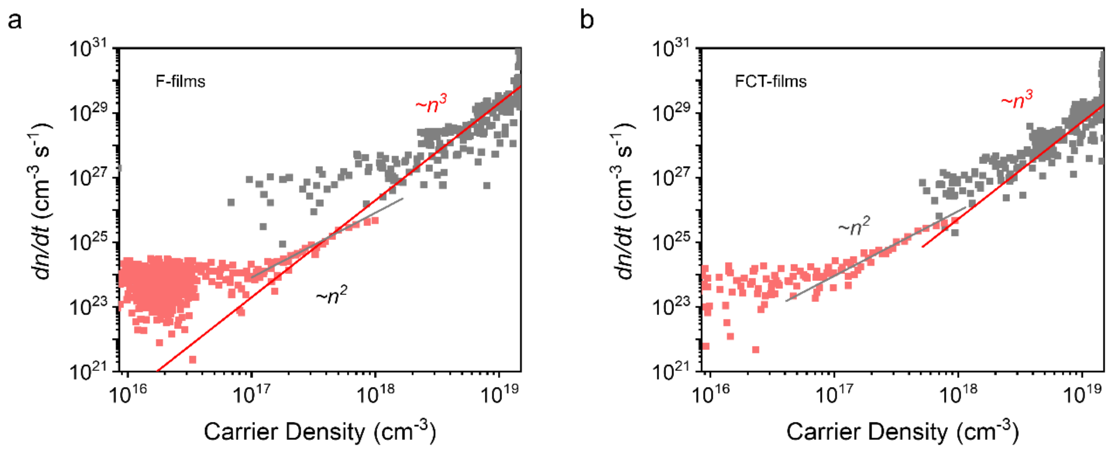


**Supplementary Figure 9. Recombination rate measurements of perovskite films.** Recombination rate over charge density of F-films (**a**) and FCT-films (**b**). The constant values were extracted from TA measurements for two different fluences with each symbol representing one measurement. The normalized TA kinetics were multiplied with the initial charge carrier density $n_0$ (determined from fluence and absorption values), and taking the time derivative of this carrier density $n(t)$. We observe different scaling of the recombination rate dn/dt with $n$ indicating transitions between different recombination mechanisms. The solid lines are fitting curves.

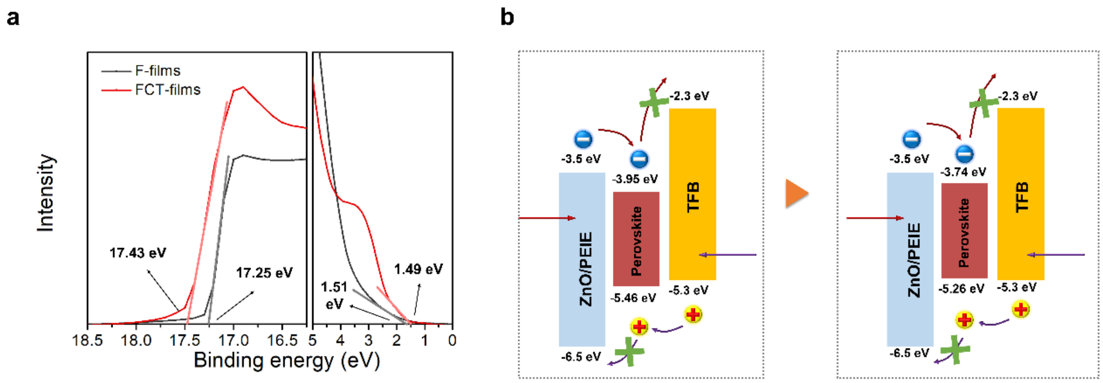


**Supplementary Figure 10. Determining the evolution of energy levels of perovskites**. **a**, UPS spectra of the F- and FCT- perovskite films (showing the secondary electron cut-off ($E_{cut\text{-}off}$) and the ionization edge. The binding energy is referenced with respect to the Fermi level of the system. He-Iα = 21.22 eV). **b**, The flat-band energy level diagram of the F- (left) and FCT-(right) device components. The energy levels of TFB and ZnO are from literatures.[15] The energy levels of conduction bands of perovskites were determined by UPS and optical bandgap from UV-Vis absorption spectra in Supplementary Figure 17b.

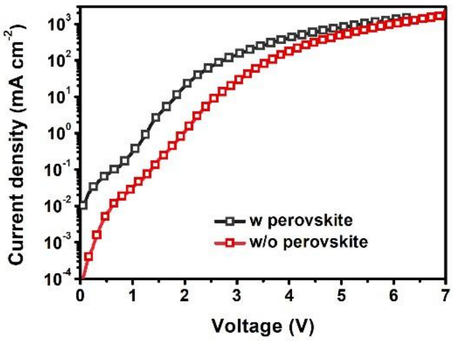


**Supplementary Figure 11. The evidence for the electrical shunts under high excitation.** Current density vs voltage curves of the FA-2.4 device and ITO/ZnO/PEIE/TFB/$MoO_3$/Au device.

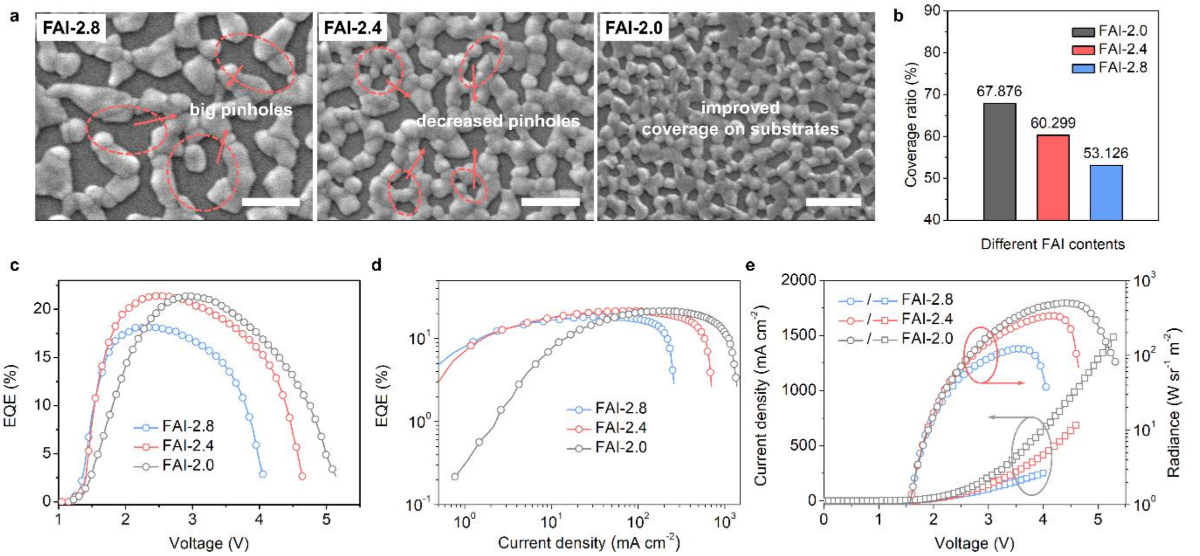


**Supplementary Figure 12. Correlation between surface coverage and device performance. a**, SEM images of FAI-2.8, FAI-2.4, and FAI-2.0 perovskite films. The scale bar represents 1 μm. **b**, The schematic diagram of device coverage. **c-e**, Characteristics of PeLEDs with different FAI contents: EQE *vs* voltage curves (**c**), EQE *vs* current density curves (**d**), and current density/radiance *vs* voltage curves (**e**).

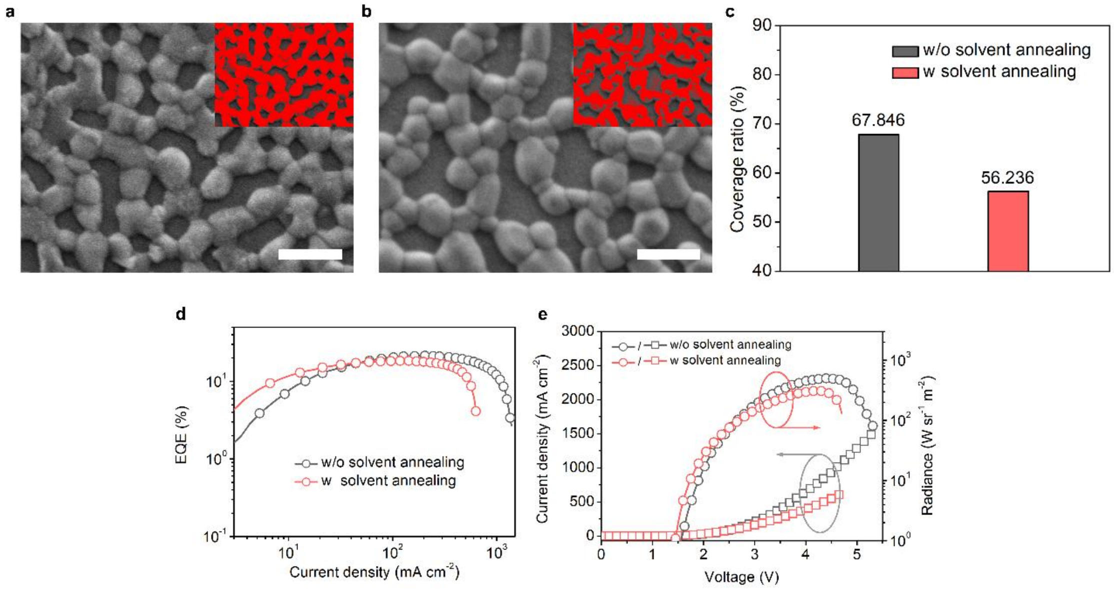


**Supplementary Figure 13. Understanding the role of perovskite film morphology on device performance.** SEM images of FAI-2.0 perovskite films without (**a**) and with (**b**) solvent annealing. The scale bar represents 500 nm. **c**, The schematic diagram of device coverage. **d**, EQE *vs* current density curves of FAI-2.0 perovskite films without and with solvent annealing. **e**, Current density/radiance *vs* voltage curves of FAI-2.0 perovskite films without and with solvent annealing.

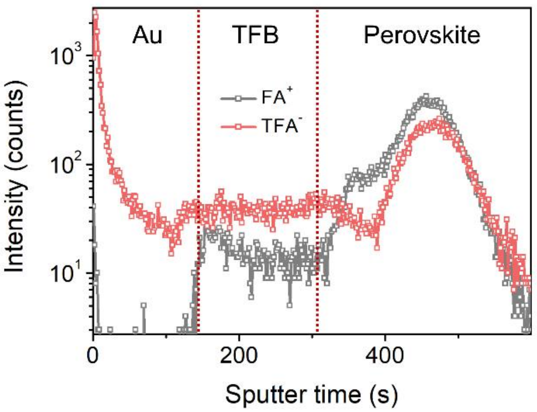


**Supplementary Figure 14. The evidence for TFA in perovskite films.** ToF-SIMS measurements of F- and FCT-PeLEDs.

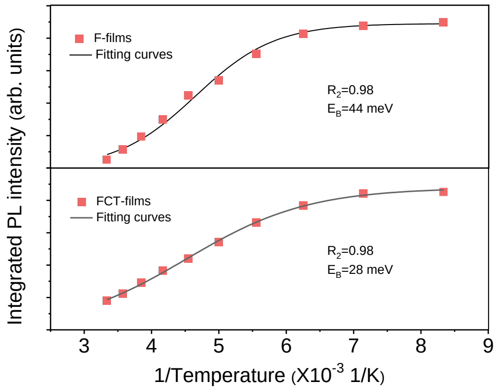


**Supplementary Figure 15. PL characteristics of the films.** Integrated PL emission intensity as a function of temperature for F- and FCT-films.

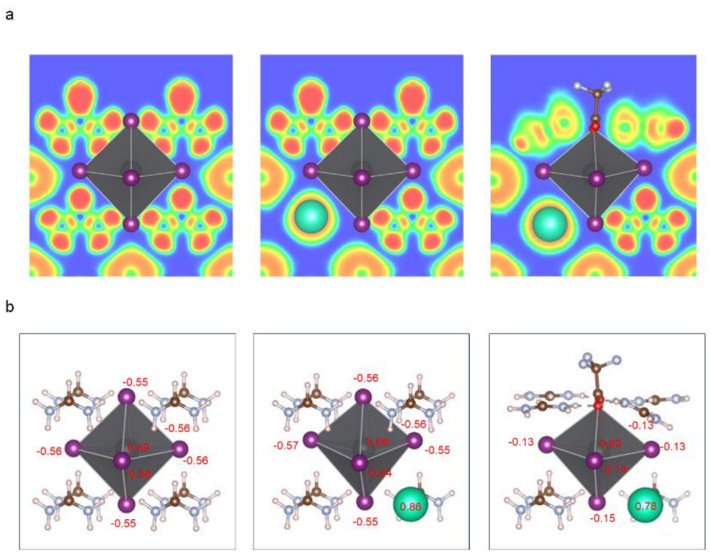


**Supplementary Figure 16. Electron localization function (ELF) and effective Bader charge.** ELF (a) and effective Bader charge (b) for $FAPbI_3$, $FA_{1-x}Cs_xPbI_3$, and $FA_{1-x}Cs_xPbI_3$ with $TFA^-$ adsorption, respectively.

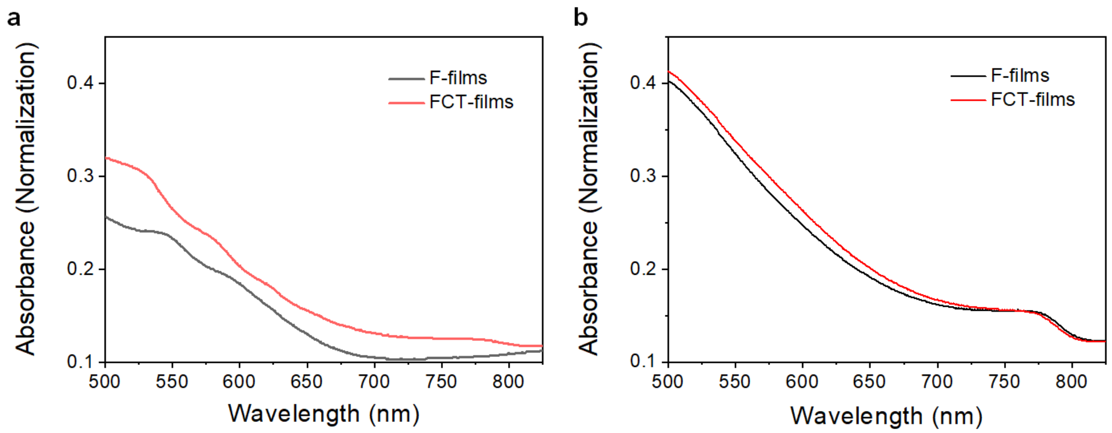


**Supplementary Figure 17. Absorption measurements of perovskite films before annealing.** UV-Vis absorption of the F- and FCT-perovskite precursor films before (**a**) and after (**b**) annealing. The absorption spectra show the incorporation of $TFA^{-}$ into perovskite precursors facilitated the perovskite formation before annealing.

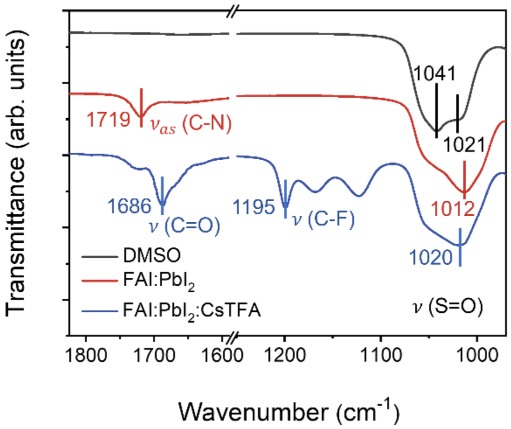


**Supplementary Figure 18. The evidence for the interactions of TFA with perovskite precursors.** ATR-FTIR spectroscopy of DMSO, FAI: $PbI_2$: DMSO, and FAI: $PbI_2$: CsTFA: DMSO. $\nu$: Stretching vibration. $\nu_{as}$: Antisymmetric stretching vibration.

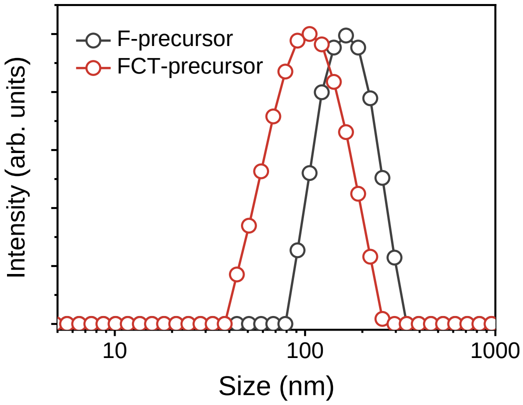


**Supplementary Figure 19. Dynamic light scattering (DLS) measurements of precursor solution.** Size distribution of control and target solutions by DLS.

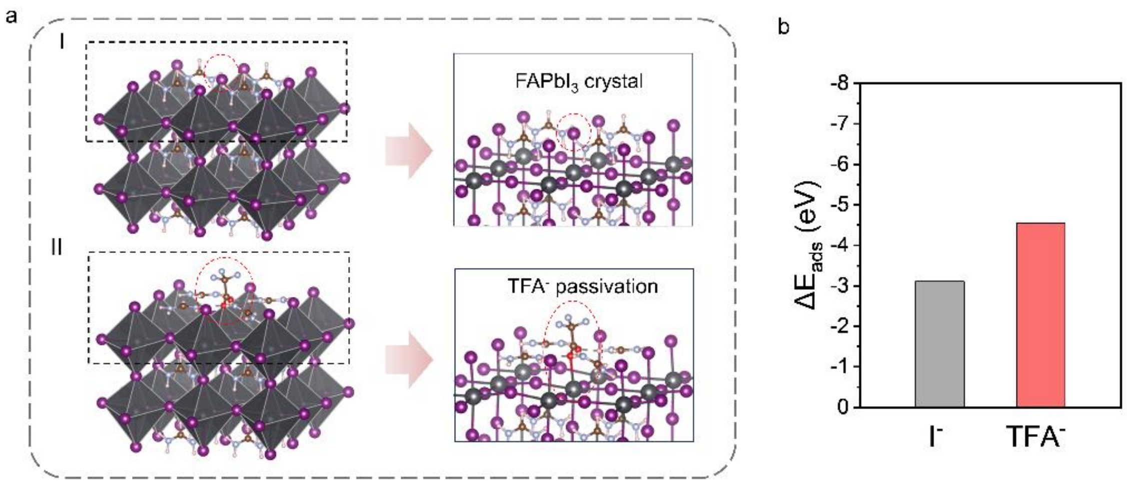


**Supplementary Figure 20. Understanding the effects of TFA on perovskite crystals. a**, Lattice structure and the adsorption of the $I^-$ and $TFA^-$ from the perovskite. **b**, The adsorption energy of the $I^-$ and $TFA^-$ on the perovskite crystals.

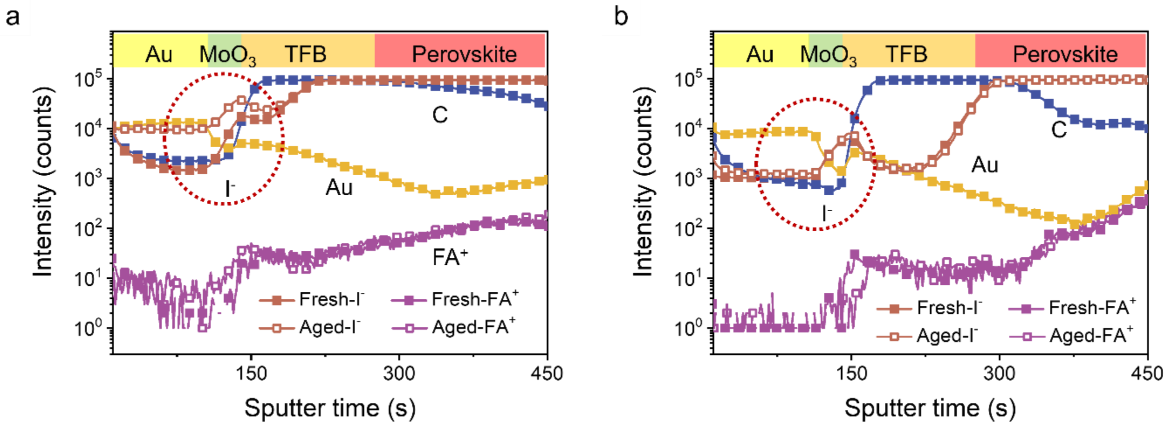


**Supplementary Figure 21. The evidence for ion migration.** ToF-SIMS of fresh device and aged device for F- (**a**) and FCT- (**b**) PeLEDs.

**Supplementary Table 1 A comparison of the key parameters with previously reported PeLEDs**

| Time | Emitter | Peak position (nm) | Peak EQE (%) | Peak radiance (W sr$^{-1}$ m$^{-2}$) | Operational current density (mA cm$^{-2}$) | T50 stability (h) | Ref. |
|---|---|---|---|---|---|---|---|
| 2018 | PPBH | 795 | 20.1 | -- | 0.1 | 46 | 1 |
| 2018 | $FAPbI_3$ | 803 | 20.7 | 390 | 100 | 20 | 2 |
| 2019 | $FAPbI_3$ | 802 | 14.2 | 241 | 100 | 23.7 | 3 |
| 2019 | $Cs_{0.1}FA_{0.9}PbI_3$ | 780 | 19.6 | 301.8 | 20 | 7 | 4 |
| 2019 | $FAPbI_3$ | 800 | 21.6 | 308 | 25 | 20 | 5 |
| 2019 | 2D $FAPbI_3$-DJ | 785 | 5.2 | 88.5 | 25 | 100 | 6 |
| 2020 | $FAPbI_3$ | 802 | 17.3 | ~200 | 20 | 100 | 7 |
| 2020 | $FA_{0.83}Cs_{0.17}PbI_3$ | 780 | 15.01 | 886.6 | 100 | 11.3 | 8 |
| 2021 | $FAPbI_3$ | 802 | 18.6 | 334 | 20 | 682 | 9 |
| 2021 | $FAPbI_3$ | 800 | 22.2 | 250 | 100 | 18.6 | 10 |
| 2021 | $FA_{0.83}Cs_{0.17}PbI_3$ | 789 | 17.5 | 1,282.7 | 100 | 130 | 11 |
| 2021 | $FAPbI_3$ | 800 | 22.8 | -- | 100 | 2.14 (T70) | 12 |
| 2022 | $FAPbI_3$ | 800 | 22.8 | 278.9 | 100 | 120.3 | 13 |
| 2023 | $FAPbI_3$ | 800 | 23.8 | 666.3 | 100 | 32 | 14 |
| 2023 | $FAPbI_3$ | 800 | 25.5 | 487 | 62.5 | 15 (T75) | 15 |
| 2024 | $FAPbI_3$ | 800 | 23.6 | 964 | 100 | 106.1 | 16 |
| 2024 | $FA_{0.83}Cs_{0.17}PbI_3$ | 789 | 23.2 | 1593 | 100 | 227 | 17 |
| **2024** | $FA_{0.88}Cs_{0.12}PbI_3$ | 786 | 21.4 | 2,409 | 100 | 142 | This work |

**Supplementary Table 2** Recombination constants of perovskite thin films. The monomolecular ($k_1$), bimolecular ($k_2$), and trimolecular ($k_3$) recombination constant and lifetimes ($\tau$).

| | | $k_1$ ($s^{-1}$) | $k_2$ ($cm^3\ s^{-1}$) | $k_3$ ($cm^6\ s^{-1}$) | $\tau$ (ns) |
|---|---|---|---|---|---|
| F-films | TRPL | $3.57\times10^{6}\pm0.28\times10^{6}$ | $3.84\times10^{-10}\pm2.69\times10^{-11}$ | $3.58\times10^{-28}\pm1.71\times10^{-29}$ | 818 |
| | TA | | $3.11\times10^{-10}\pm2.84\times10^{-11}$ | $2.98\times10^{-28}\pm3.13\times10^{-29}$ | |
| FCT-films | TRPL | $1.30\times10^{6}\pm0.13\times10^{6}$ | $3.10\times10^{-10}\pm1.10\times10^{-11}$ | $1.15\times10^{-28}\pm6.39\times10^{-30}$ | 1112 |
| | TA | | $2.28\times10^{-10}\pm2.76\times10^{-11}$ | $5.28\times10^{-29}\pm4.31\times10^{-30}$ | |

The average values were derived from four measurements of different samples.

## Supplementary References


1. Zhao, B. et al. High-efficiency perovskite–polymer bulk heterostructure light-emitting diodes. *Nat. Photon.* **12**, 783–789 (2018).
2. Cao, Y. et. al. Perovskite light-emitting diodes based on spontaneously formed submicrometre-scale structures. *Nature* **562**, 249-253 (2018).
3. Miao, Y. et al. Stable and bright formamidinium-based perovskite light-emitting diodes with high energy conversion efficiency. *Nat. Commun.* **10**, 1–7 (2019).
4. Yuan, Z. et. al. Unveiling the synergistic effect of precursor stoichiometry and interfacial reactions for perovskite light-emitting diodes. *Nat. Commun.* **10**, 2818 (2019).
5. Xu, W. et. al. Rational molecular passivation for high-performance perovskite light-emitting diodes. *Nat. Photon.* **13**, 418-424 (2019).
6. Shang, Y. et. al. Highly stable hybrid perovskite light-emitting diodes based on Dion-Jacobson structure. *Sci. Adv.* **5**, 8072 (2019).
7. Wang, H. et al. Perovskite-molecule composite thin films for efficient and stable light-emitting diodes. *Nat. Commun.* **11**, 1–9 (2020).
8. Guo, Y. et al. Degradation mechanism of perovskite light-emitting diodes: an in-situ investigation via electroabsorption spectroscopy and device modelling. *Adv. Funct. Mater.* **30**, 1910464 (2020).
9. Kuang, C. et al. Critical role of additive-induced molecular interaction on the operational stability of perovskite light-emitting diodes. *Joule* **5**, 618-630 (2021).
10. Zhu, L. et. al. Unveiling the additive-assisted oriented growth of perovskite crystallite for high performance light-emitting diodes. *Nat. Commun.* **12**, 5081 (2021).
11. Guo, Y. et. al. Phenylalkylammonium passivation enables perovskite light emitting diodes with record high-radiance operational lifetime: the chain length matters. *Nat. Commun.* **12**, 644 (2021).
12. Teng, P. et al. Degradation and self-repairing in perovskite light-emitting diodes. *Matter*, **4**, 3710-3724 (2021).
13. Guo, B. et. al. Ultrastable near-infrared perovskite light-emitting diodes. *Nat. Photon.* **16**, 637-643 (2022).
14. Sun, Y. et al. Bright and stable perovskite light-emitting diodes in the near-infrared range. *Nature* **615**, 830-835 (2023).
15. Li, J. et al. Self-generated buried submicrocavities for high-Performance near-Infrared perovskite light-emitting diode. *Nano-Micro Lett.* **15**, 125 (2023).
16. Li, Z. et al. Eliminating the Adverse Impact of Composition Modulation in Perovskite Light-Emitting Diodes toward Ultra-High Brightness and Stability. *Adv. Mater.* **36**, 2313981 (2024).

17. Li, Z. et al. Grain orientation management and recombination suppression for ultra-stable PeLEDs with record brightness. *Joule*, **8**, 1176-1190 (2024).
